# Rethinking Congestion in Heart Failure from Volume Overload to Venous Pressure and Organ Disfunction with VExUS

**DOI:** 10.3390/medicina62071224

**Published:** 2026-06-24

**Authors:** Marcello Marchetta, Lucio Giuseppe Granata, Anna Rosa Napoli, Fabiana Cipolla, Giuseppe Massimo Sangiorgi, Giuseppina Maura Francese, Simona Giubilato

**Affiliations:** 1 Cardiology Division, Tor Vergata Hospital, 00133 Rome, Italy; marcello.marchetta1997@gmail.com (M.M.); g.sangiorgi@gmail.com (G.M.S.); 2 Institute of Cardiovascular Disease of Sremska Kamenica, 21204 Novi Sad, Serbia; 3 Cardiology Division, Garibaldi-Nesima Hospital, ARNAS Garibaldi, 95122 Catania, Italy; 4 Cardiology Unit, Department of Clinical and Experimental Medicine, University Hospital “G. Martino”, University of Messina, 98122 Messina, Italy; annarosanapoli1510@gmail.com (A.R.N.); fabiana.cipolla@libero.it (F.C.); 5 Cardiology Division, Cannizzaro Hospital, 95126 Catania, Italy; simogiub@hotmail.com

**Keywords:** acute heart failure, heart failure, venous congestion, VExUS, point-of-care ultrasound, cardiorenal syndrome, intrarenal venous Doppler, lung ultrasound, decongestive therapy, diuretic

## Abstract

Congestion is a major driver of symptoms, hospitalization, and adverse outcomes in heart failure (HF), yet its clinical assessment remains challenging. Traditional approaches based on physical examination, biomarkers, and isolated imaging surrogates often fail to capture the complexity of systemic venous congestion and its impact on organ function. In HF, congestion should be interpreted as a multifactorial process resulting from the interaction between intravascular volume burden, venous compliance, cardiac filling pressures, neurohormonal activation, blood volume redistribution, and organ-specific susceptibility. In this context, point-of-care ultrasound has emerged as a promising adjunctive tool for bedside congestion assessment. The Venous Excess Ultrasound (VExUS) score integrates inferior vena cava assessment with Doppler analysis of hepatic, portal, and intrarenal veins, allowing for the evaluation of venous pressure transmission and organ-level congestion. Observational studies suggest that VExUS and related venous Doppler abnormalities correlate with invasive hemodynamic parameters and are associated with acute kidney injury, diuretic response, heart failure hospitalization, and mortality. Serial changes in venous congestion may provide additional information regarding treatment response and clinical trajectory. However, the available evidence remains heterogeneous across acute HF, ambulatory HF, cardiorenal syndrome, and critical care populations, and randomized trials evaluating VExUS-guided management are lacking. Therefore, VExUS should be interpreted as a complementary tool within a multimodal assessment that includes echocardiography, lung ultrasound, biomarkers, renal function, urine output, physical examination, and response to therapy. By integrating fluid burden with venous pressure transmission and organ perfusion, multimodal ultrasound may support more individualized congestion assessment and risk stratification in HF.

## 1. Introduction

Heart failure (HF) remains a leading cause of morbidity and hospitalization worldwide, with congestion representing the primary driver of symptoms, disease progression, and adverse outcomes [1]. In patients hospitalized for acute HF, achieving complete decongestion remains challenging, with studies suggesting that more than 50% of patients still exhibit residual congestion at the time of discharge despite apparent clinical stabilization [2]. Despite advances in pharmacological and device-based therapies, the persistence of congestion continues to account for a substantial proportion of rehospitalizations and mortality [3]. Notably, residual congestion at the time of discharge has been consistently associated with worse clinical outcomes, even in patients deemed clinically stable [4].

Traditionally, the assessment of congestion in HF has relied on a combination of clinical examination, invasive hemodynamic measurements, and surrogate imaging or biochemical markers. However, each of these approaches carries significant limitations [5]. Physical examination lacks sensitivity and reproducibility, often failing to detect early or subclinical congestion. Invasive measures such as central venous pressure (CVP) provide only a partial representation of systemic hemodynamics and do not reliably reflect organ-level congestion. Similarly, echocardiographic surrogates such as inferior vena cava (IVC) diameter offer limited specificity, while biomarkers capture cardiac wall stress but do not directly quantify venous congestion or its impact on end-organ function [6,7].

These limitations highlight a fundamental issue in contemporary HF management: the incomplete characterization of venous congestion. Congestion is often conceptualized as a simple excess of intravascular volume, yet accumulating evidence suggests a far more complex pathophysiological process. Venous congestion reflects the interplay between cardiac function, venous return, organ-specific drainage, intravascular volume status, venous compliance, and neurohormonal activation, ultimately leading to elevated venous pressures and impaired organ perfusion. Although venous congestion may occur even in the absence of overt fluid accumulation, volume overload and venous pressure transmission frequently coexist in clinical HF and should be viewed as complementary rather than mutually exclusive mechanisms. Conventional diagnostic tools often fail to adequately capture this complexity [8,9].

In this context, point-of-care ultrasound (POCUS) has emerged as a promising modality for the bedside assessment of congestion. Among the available techniques, the Venous Excess Ultrasound (VExUS) score represents a novel, integrative approach that combines Doppler assessment of the IVC, hepatic, portal, and intrarenal veins to quantify systemic venous congestion [10]. By directly evaluating venous flow patterns across multiple organ systems, VExUS provides a physiologically grounded assessment of congestion that extends beyond traditional measures [11].

VExUS has been shown to correlate with invasive hemodynamic parameters, including right atrial pressure, and to identify patients at increased risk of adverse outcomes, particularly acute kidney injury and rehospitalization [12,13]. Furthermore, dynamic changes in VExUS over time appear to carry greater prognostic significance than single measurements, underscoring the importance of serial assessment in clinical practice [14]. These observations support a paradigm shift from a static, volume-centered view of congestion to a dynamic, physiology-based one driven by venous pressure and organ-level dysfunction.

This review aims to provide a pathophysiological and clinically oriented framework for understanding venous congestion in HF, with a particular focus on the transition from volume-centered to organ-centered congestion assessment. Rather than providing a purely technical overview of VExUS, this review integrates current evidence on venous pressure transmission, organ-specific dysfunction, cardiorenal interactions, and multimodal ultrasound assessment into a unified conceptual model. The objective is to contextualize VExUS within the broader pathophysiology of heart failure and to discuss its potential role as a tool for organ-relevant congestion assessment and clinical decision support.

## 2. Materials and Methods

A structured literature search was performed using PubMed/Medline to identify relevant studies from inception to 31 March 2026, without any language restriction. The search strategy included combinations of keywords such as “heart failure”, “venous congestion”, “VExUS”, “point-of-care ultrasound”, “cardiorenal syndrome”, “renal Doppler”, “lung ultrasound”. Additional references were identified through manual screening of the bibliographies of selected articles.

Eligible publications included original research articles, observational cohort studies, systematic reviews, meta-analyses, consensus documents, and guideline papers addressing the pathophysiology, diagnostic assessment, prognostic implications, and therapeutic relevance of venous congestion in HF.

Particular emphasis was placed on studies evaluating Doppler-based ultrasound techniques, including VExUS, intrarenal venous Doppler, portal vein Doppler, and multimodal ultrasound assessment of congestion. Studies not directly related to heart failure, venous congestion, or ultrasound-based congestion assessment were excluded. Small exploratory studies and abstract-only publications were considered only when providing clinically relevant or emerging mechanistic insights. Priority was given to contemporary evidence reflecting current clinical practice, evolving concepts of congestion physiology, and integration with modern HF management strategies.

Given the narrative nature of the review, no formal meta-analytic techniques or quantitative pooling of results were undertaken. Particular emphasis was placed on studies exploring the relationship between venous pressure transmission, organ dysfunction, and ultrasound-based congestion assessment. The available evidence was synthesized to provide a mechanism-based and clinically oriented framework linking ultrasound-based congestion assessment with pathophysiological mechanisms, organ dysfunction, and therapeutic implications in HF.

## 3. Rethinking Congestion: Beyond Volume Overload

For decades, congestion in heart failure has been predominantly conceptualized as a consequence of volume overload. This paradigm, although clinically intuitive, fails to capture the complexity of the underlying hemodynamic and organ-level interactions that characterize congestive states. Emerging evidence suggests that congestion is not merely the result of excess intravascular volume, but rather the expression of an altered equilibrium between cardiac function, venous return, and organ-specific drainage [15].

Importantly, this perspective should not be interpreted as opposing the traditional concept of volume overload. In HF, congestion usually results from the interaction between intravascular volume expansion, altered venous compliance, elevated filling pressures, neurohormonal activation, and blood volume redistribution. Rather than replacing the concept of fluid overload, the venous congestion paradigm highlights that the clinical consequences of congestion are determined not only by the amount of retained fluid but also by the way pressure is transmitted through the venous circulation and affects end-organ function [16].

A central determinant of congestion is right atrial pressure, which reflects the downstream resistance encountered by venous return [17]. Elevated right atrial pressure increases systemic venous pressure and is transmitted retrogradely to the venous circulation of multiple organs, including the kidneys, liver, and splanchnic system. This retrograde transmission of pressure, rather than absolute volume, represents a key driver of organ dysfunction [8,18].

In this context, the concept of retrograde venous afterload provides a useful conceptual approach to understand the pathophysiology of congestion.

As venous pressures rise, the pressure gradient between arterial inflow and venous outflow is reduced, leading to impaired organ perfusion despite preserved or only mildly reduced cardiac output [19]. This mechanism is particularly evident at the renal level, where increased venous pressure directly reduces renal perfusion pressure and glomerular filtration, independently of systemic arterial hypotension [8,20].

Venous congestion may occur even in the absence of overt fluid overload.

Clinical and experimental studies have demonstrated that patients with similar degrees of intravascular volume expansion may exhibit markedly different levels of congestion depending on cardiac function, venous compliance, neurohormonal activation, and redistribution of blood volume within the splanchnic compartment [15,21].

Experimental and translational observations suggest that acute shifts in venous capacitance may substantially increase cardiac filling pressures even in the absence of major changes in total body fluid volume, supporting the concept that congestion is not solely a marker of fluid accumulation but rather a complex hemodynamic state [21,22].

In routine clinical practice, this may explain why patients with apparently comparable volume status often present with markedly different degrees of organ dysfunction and clinical decompensation.

This evolving pathophysiological framework is increasingly reflected in contemporary HF management strategies and guideline-oriented approaches emphasizing comprehensive congestion assessment beyond simple estimation of fluid overload [1].

These considerations challenge the traditional dichotomy between “volume overload” and “euvolemia,” suggesting that congestion should instead be interpreted as a hemodynamic state characterized by elevated venous pressures and impaired venous drainage. In line with this view, studies have shown that markers of venous congestion, rather than cardiac output, are more closely associated with organ dysfunction, particularly acute kidney injury [18].

Recent data suggest that venous congestion assessed by ultrasound may reflect underlying cardiac function more than intravascular volume alone. In critically ill populations, VExUS has been shown to correlate with cardiac performance parameters, supporting the concept that congestion represents the downstream manifestation of cardiac dysfunction rather than a purely volumetric phenomenon [23].

Another key aspect of congestion is its interaction with systemic perfusion. The relationship between perfusion and congestion is not mutually exclusive but deeply interconnected. Patients may simultaneously exhibit elevated venous pressures and reduced forward flow, resulting in a combined state of congestion and hypoperfusion [24].

This interaction has important clinical implications, as therapies targeting only one component, such as diuretics for volume reduction or inotropes for cardiac output, may fail to adequately address the underlying pathophysiology.

Finally, the dynamic nature of congestion must be emphasized. Congestion is not a static condition but a fluctuating process that evolves in response to treatment and disease progression [16]. Changes in congestion over time, assessed through serial ultrasound evaluation, have been shown to carry greater prognostic significance than single timepoint measurements, highlighting the limitations of snapshot-based assessment [14,25].

These observations support a refinement in the conceptualization of congestion in HF: from a predominantly volume-centered model to a dynamic, multidimensional paradigm in which fluid accumulation, venous pressure transmission, organ-level dysfunction, and systemic perfusion interact to determine clinical manifestations and outcomes.

The key differences between traditional and contemporary conceptualizations of congestion are summarized in Table 1.

**Figure 1:**
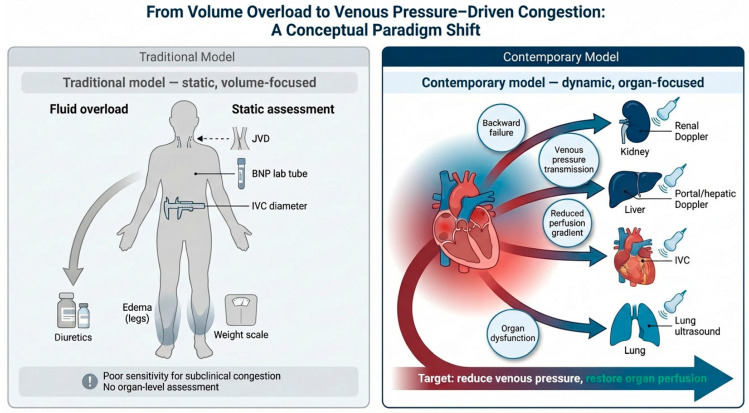
From volume overload to venous pressure-driven congestion. Traditional models conceptualize congestion as intravascular fluid accumulation assessed through clinical and surrogate markers. However, this approach fails to capture subclinical and organ-level congestion. Contemporary evidence supports a physiology-based paradigm in which congestion is driven by elevated venous pressures and impaired venous drainage. Retrograde transmission of right atrial pressure affects peripheral organs, reducing the effective perfusion gradient and contributing to organ dysfunction independently of cardiac output. Multimodal ultrasound, including VExUS, enables direct assessment of venous congestion and supports a more precise and clinically relevant evaluation. Abbreviations: JVD, jugular vein distension; BNP, brain natriuretic peptide; IVC, inferior cava vein.

The transition from a volume-centered interpretation of congestion to a dynamic model based on venous pressure transmission and organ dysfunction is schematically summarized in Figure 1.

## 4. The Concept of Organ-Relevant Congestion

A critical limitation of traditional approaches to congestion lies in the implicit assumption that all forms of congestion are clinically equivalent. In reality, increasing evidence indicates that not all congestion is clinically relevant, and that its clinical impact is largely determined by its effects at the level of individual organs [26].

Among the various organ systems involved, the kidney represents the most extensively studied and clinically relevant target of venous congestion. In the context of acute heart failure, renal dysfunction has traditionally been attributed to reduced cardiac output and impaired arterial perfusion.

However, this paradigm has been increasingly challenged by data demonstrating that venous congestion plays a central, and often dominant, role in the development of acute kidney injury [18,20].

Intrarenal Doppler studies have provided key insights into this mechanism. Under normal conditions, renal venous flow is continuous, reflecting unobstructed venous drainage [26].

In contrast, elevated right atrial and systemic venous pressures lead to progressive alterations in intrarenal venous flow patterns, ranging from biphasic to monophasic discontinuous flow [27].

These abnormalities reflect increasing renal venous stasis and congestion and have been shown to correlate with both the presence and severity of acute kidney injury and, importantly, occur independently of cardiac output.

In patients with acute decompensated heart failure, abnormal intrarenal venous flow patterns have been observed despite similar cardiac index values, highlighting that renal dysfunction may arise primarily from impaired venous outflow rather than reduced arterial inflow [20].

This observation fundamentally shifts the understanding of cardiorenal syndrome, emphasizing the role of backward pressure transmission rather than forward flow limitation.

The pathophysiological consequences of venous congestion on renal function can be interpreted as a reduction in the effective renal perfusion gradient, defined as the difference between arterial inflow and venous outflow pressure. As venous pressure rises, this gradient progressively narrows, impairing glomerular filtration despite preserved systemic arterial pressure or only mildly reduced cardiac output. Therefore, venous congestion should be regarded as one important contributor to renal dysfunction rather than the sole explanatory mechanism [10,24].

**Figure 2:**
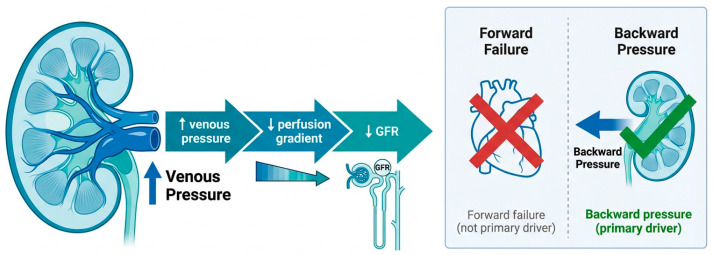
Venous congestion as the primary driver of renal dysfunction in heart failure: the role of impaired perfusion gradient. Renal dysfunction in heart failure has traditionally been attributed to reduced cardiac output and impaired arterial perfusion (“forward failure”). However, accumulating evidence supports a dominant role of venous congestion in the pathophysiology of cardiorenal syndrome. Elevated right atrial and systemic venous pressures are transmitted retrogradely to the renal venous circulation, increasing renal interstitial pressure and reducing the effective renal perfusion gradient, defined as the difference between arterial inflow and venous outflow pressure. Abbreviation: GFR, glomerular filtration rate.

This mechanism, illustrated in Figure 2, further supports the concept that renal dysfunction in heart failure is often driven by backward pressure transmission rather than by reduced forward flow.

The concept of congestive nephropathy further reinforces this perspective and provides a pathophysiological link between systemic venous congestion and cardiorenal syndrome.

Congestive nephropathy has been proposed as a distinct congestion-driven phenotype of renal dysfunction, characterized by elevated venous pressures, impaired renal venous outflow, increased renal interstitial pressure, and reduced effective renal perfusion gradient. This paradigm is supported by clinical studies showing that venous congestion, rather than reduced cardiac output alone, is a major determinant of worsening renal function in decompensated heart failure [28,29]. In parallel, multimodal approaches integrating ultrasound and biomarkers have identified a substantial proportion of patients with congestion-driven renal impairment, supporting the clinical relevance of this entity [30].

Another important implication is the temporal dissociation between organ congestion and conventional markers of organ dysfunction. Structural and hemodynamic alterations at the organ level often precede measurable changes in laboratory parameters [31]. In particular, abnormal intrarenal venous flow patterns may be detected before any rise in serum creatinine, indicating that organ congestion represents an early and potentially reversible stage of injury [32].

This has direct clinical consequences. Reliance on delayed markers such as creatinine may lead to under-recognition of early congestion-related injury and inappropriate therapeutic decisions. In clinical practice, worsening renal function is often interpreted as a consequence of hypoperfusion, prompting reduction or discontinuation of diuretic therapy. However, in the presence of persistent venous congestion, reducing or withholding decongestive therapy solely because of worsening renal function may be inappropriate, as it may leave the main hemodynamic driver of renal dysfunction untreated [29]. Current expert recommendations on diuretic therapy in HF emphasize that renal function changes should be interpreted in the context of the overall decongestive response, rather than in isolation. In this setting, Doppler-based assessment of venous congestion may help distinguish congestion-related renal dysfunction from true hypoperfusion and support more individualized diuretic management [20,33].

Beyond the kidney, similar principles apply to other organ systems. Hepatic and splanchnic congestion, reflected by alterations in portal and hepatic venous flow, represent clinically relevant non-renal manifestations of systemic venous hypertension. Portal vein pulsatility has been associated with right-sided filling pressures and adverse outcomes in patients with HF, supporting its role as a marker of abdominal venous congestion. Beyond hepatic dysfunction, splanchnic congestion may contribute to intestinal edema, impaired drug absorption, systemic inflammation, and reduced abdominal organ perfusion [34,35]. The splanchnic compartment, in particular, acts as a dynamic reservoir that modulates venous return and central pressures, further influencing the development and progression of congestion [9].

The clinical relevance of congestion extends beyond the acute setting. In ambulatory HF populations, subclinical venous congestion detectable by ultrasound-based techniques, including VExUS, has been associated with worse short-term outcomes, including heart failure hospitalization and renal deterioration. These findings suggest that clinically silent congestion may represent an underrecognized therapeutic target across the spectrum of heart failure and may allow earlier therapeutic optimization before overt decompensation occurs [36].

These findings suggest that clinically silent congestion may represent an underrecognized therapeutic target across the spectrum of heart failure. All these observations support a shift toward an organ-centered model of congestion, in which the clinical significance of congestion is determined by its impact on organ function rather than by global estimates of volume status. In this construct, the assessment of venous congestion should aim to identify early, organ-specific alterations that precede overt dysfunction and guide targeted therapeutic interventions. This perspective provides a strong rationale for the use of Doppler-based ultrasound techniques, including VExUS and intrarenal venous assessment, which enable direct visualization of congestion at the organ level and allow for a more precise characterization of its clinical relevance.

## 5. VExUS: Technical Principles and Interpretation

The VExUS score is a structured POCUS protocol designed to assess and quantify systemic venous congestion and its impact on peripheral organs. By integrating morphological assessment of the IVC with Doppler evaluation of multiple venous territories, VExUS provides a comprehensive evaluation of venous congestion at both central and organ levels [37].

From a technical standpoint, ultrasound acquisition should ideally be performed at end-expiration in order to minimize the influence of intrathoracic pressure variations on venous return and Doppler signals [38]. When feasible, simultaneous electrocardiographic monitoring may facilitate accurate temporal interpretation of venous Doppler waveforms, particularly in distinguishing systolic and diastolic components [8].

### 5.1. Inferior Vena Cava

The assessment of the IVC represents the initial step of the VExUS protocol. Measurement of IVC diameter is typically performed in the subcostal view, and a diameter greater than 2 cm and/or reduced inspiratory collapsibility < 50% suggests elevated right atrial pressure [39].

However, reliance on IVC diameter alone is limited by multiple confounding factors that may affect its accuracy independently of intravascular volume status.

These include respiratory mechanics, particularly in patients undergoing positive pressure ventilation, as well as vigorous inspiratory efforts, which can significantly alter venous return. In addition, conditions such as cirrhosis, portal hypertension, and significant tricuspid regurgitation may influence IVC size and collapsibility independently of systemic congestion [40,41,42]. These limitations highlight the need for complementary assessment of downstream venous flow.

### 5.2. Hepatic Veins

Hepatic vein Doppler interrogation is typically performed using subcostal or lateral acoustic windows. Under normal conditions, hepatic venous flow is pulsatile and reflects right atrial pressure variations, with a predominant systolic (S) wave over the diastolic (D) component [8]. With increasing venous congestion, the systolic component progressively decreases, eventually becoming smaller than the diastolic wave, and in advanced stages may reverse direction entirely.

These changes reflect the direct transmission of elevated right atrial pressure to the hepatic venous system. Interpretation may be limited in the presence of conditions such as severe tricuspid regurgitation, atrial fibrillation, or intrinsic liver disease, which can independently alter Doppler morphology [43].

### 5.3. Portal Vein

Portal vein flow is normally continuous and minimally pulsatile. In the presence of systemic venous congestion, right atrial pressure oscillations are transmitted to the splanchnic circulation, resulting in increased pulsatility of portal flow. This phenomenon can be quantified using the pulsatility index, calculated as (Vmax − Vmin)/Vmax × 100% [43]. Progressive increases in pulsatility reflect worsening congestion, although interpretation must consider confounding factors. Advanced cirrhosis may alter portal flow patterns independently of systemic congestion [44]. Furthermore, portal vein pulsatility is influenced by a complex interplay between cardiac function, splanchnic capacitance, and volume status, and may therefore be observed even in non-congested states under specific physiological conditions [22,23,38].

### 5.4. Intrarenal Venous Doppler

Intrarenal venous Doppler provides a direct assessment of the impact of venous congestion on renal hemodynamics. Examination is typically performed using a lateral approach at the mid-axillary line, targeting interlobar veins, which provide optimal Doppler alignment [40,45].

In euvolemic conditions, intrarenal venous flow is continuous and monophasic. With increasing venous congestion, flow becomes biphasic and discontinuous, reflecting partial impedance to venous outflow. In severe congestion, systolic flow may disappear entirely, resulting in a discontinuous monophasic pattern with flow limited to the diastolic phase [37].

Interpretation of renal venous flow is influenced by both systemic and local factors. These include renal interstitial edema, reduced vascular compliance, decreased arterial inflow, and increased intra-abdominal pressure, all of which may affect venous outflow independently of systemic volume status [46]. In addition, technical limitations related to vessel size and sampling location may affect measurement accuracy.

### 5.5. VExUS Scoring System

The final VExUS grade is determined by integrating IVC assessment with Doppler findings from the hepatic, portal, and intrarenal veins. A normal IVC diameter (≤2 cm) generally excludes significant systemic venous congestion, whereas a dilated IVC (>2 cm) prompts further evaluation using Doppler analysis [45].

In the most commonly used grading system, VExUS grade 0 corresponds to the absence of significant venous congestion, with normal IVC size and normal Doppler patterns. Grade 1 indicates mild congestion with a dilated IVC but only mild Doppler abnormalities. Grade 2 reflects moderate congestion, characterized by a dilated IVC associated with significant abnormalities in at least one Doppler territory. Grade 3 represents severe systemic venous congestion, with a dilated IVC and severe Doppler abnormalities involving multiple venous territories, including systolic reversal in the hepatic veins, marked portal pulsatility, and severely discontinuous intrarenal venous flow [40].

The main technical components and Doppler interpretation of the VExUS protocol are summarized in Table 2.

**Figure 3:**
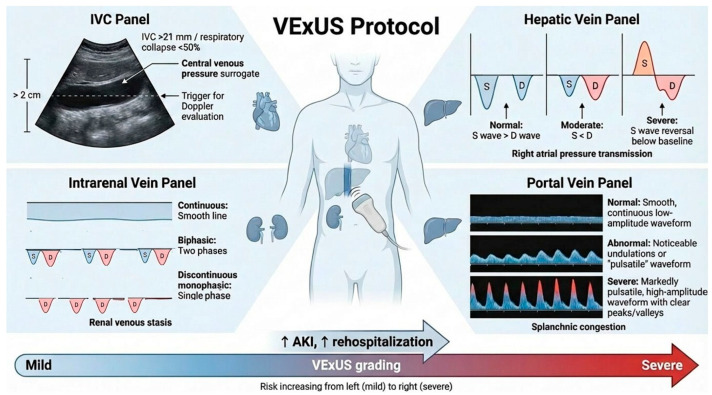
VExUS protocol and physiological interpretation. The VExUS score integrates IVC morphology with Doppler assessment of hepatic, portal, and intrarenal veins. Progressive waveform abnormalities reflect increasing venous pressure transmission and organ-level congestion. Higher VExUS grades are associated with increased risk of organ dysfunction, particularly acute kidney injury. VExUS reflects hemodynamic congestion rather than intravascular volume alone. Abbreviations: VexUS, venous excess ultrasound; IVC, inferior vena cava; S, systolic; D, diastolic; AKI, acute kidney injury.

Figure 3 provides a schematic overview of the VExUS protocol, including the main venous territories assessed and the progressive waveform abnormalities associated with increasing congestion severity.

### 5.6. Interpretation and Physiological Integration

Although initially conceptualized as a tool to assess volume status, VExUS should be interpreted within a broader hemodynamic framework. Doppler abnormalities reflect the retrograde transmission of venous pressure to peripheral organs and therefore represent markers of congestion-related organ dysfunction rather than simple intravascular volume excess [47].

Importantly, VExUS integrates multiple physiological determinants, including cardiac function, venous compliance, and intravascular volume. As a result, high VExUS grades may be observed in patients with cardiac dysfunction even in the absence of overt fluid overload, while conversely, patients with positive fluid balance may not exhibit severe congestion in the presence of preserved cardiac function [48,49].

These observations underscore the need for careful clinical interpretation. VExUS should not be viewed as a direct surrogate of volume status, but rather as a marker of the inability of the cardiovascular system to accommodate circulating volume at acceptable filling pressures [50]. Consequently, its interpretation should always be integrated with assessment of biventricular function and global hemodynamics [23].

Furthermore, individual Doppler abnormalities should always be interpreted within the broader clinical context, as several conditions may significantly influence venous flow patterns independently of intravascular volume status. These include severe tricuspid regurgitation, atrial fibrillation, pulmonary hypertension, right ventricular dysfunction, mechanical ventilation, cirrhosis with portal hypertension, chronic kidney disease, and elevated intra-abdominal pressure [9,47,51,52].

### 5.7. Simplified, Component-Based, and Extended VExUS Approaches

Recent developments have explored simplified, component-based, and extended approaches to venous congestion assessment, with the aim of improving feasibility in settings where full VExUS acquisition may be technically difficult or time-consuming.

Simplified VExUS approaches, including modified or abbreviated protocols, generally aim to reduce the number of Doppler territories assessed while preserving clinically meaningful information on systemic venous congestion. These strategies may be particularly useful in emergency departments, intensive care units, or technically challenging examinations, where rapid bedside assessment is required. However, simplified approaches may reduce the physiological completeness and specificity provided by full multi-organ VExUS assessment [47,50].

Component-based strategies focusing on individual Doppler parameters, particularly portal vein pulsatility or intrarenal venous Doppler, have also shown clinical relevance. In a prospective ICU study evaluating patients receiving loop diuretics, portal vein pulsatility and renal venous impedance predicted appropriate diuretic-induced decongestion, with portal vein pulsatility showing higher discrimination than the composite VExUS score. These findings suggest that selected Doppler components may be useful when the clinical question is focused on treatment response or when complete VExUS acquisition is not feasible [53].

Femoral venous Doppler has emerged as another simplified and easily accessible approach for assessing systemic venous congestion and right-sided filling pressures. Its practical advantages include rapid acquisition and favorable acoustic accessibility, particularly in critically ill patients. Nevertheless, current evidence remains preliminary and heterogeneous, and femoral venous Doppler should be considered complementary rather than a replacement for integrated multi-organ assessment [40].

Extended VExUS approaches, incorporating additional venous territories such as the superior vena cava, splenic vein, or femoral venous Doppler, may be useful when conventional abdominal windows are limited or when a broader assessment of systemic venous congestion is desired. However, these approaches require further validation, and standardized acquisition protocols and interpretation thresholds are still lacking [54].

The choice between full VExUS, simplified/component-based assessment, and extended approaches should depend on the clinical setting, operator expertise, acoustic feasibility, and the specific clinical question. Full VExUS remains more physiologically comprehensive, whereas simplified or alternative approaches may be preferable for rapid screening, serial monitoring, or technically limited examinations.

## 6. VExUS vs. Other Congestion Assessment Tools

The evaluation of congestion in heart failure has traditionally relied on a combination of clinical examination, imaging surrogates, and biochemical markers. However, each of these tools captures only a limited component of the congestive process, often resulting in incomplete or misleading assessments [55].

In this context, the emergence of VExUS highlights the limitations of conventional approaches and supports a more integrative evaluation of systemic venous congestion.

Clinical examination remains the first-line method for assessing congestion but is intrinsically limited by low sensitivity and significant interobserver variability. Signs such as peripheral edema, jugular venous distension, and pulmonary rales often appear late in the course of disease and may be absent in patients with subclinical congestion [56].

Importantly, a substantial proportion of patients considered clinically decongested at discharge continue to exhibit evidence of congestion when assessed with ultrasound, which is associated with a significantly increased risk of rehospitalization [4].

Among imaging tools, IVC diameter is widely used as a surrogate of right atrial pressure. Although simple and rapid to obtain, IVC assessment provides only a central estimate of venous pressure and does not reflect downstream organ congestion [57].

As a result, patients with similar IVC findings may have markedly different degrees of organ-level involvement, limiting its specificity for clinically relevant congestion [11]. Furthermore, recent data showed that venous congestion may be present even in patients with IVC diameters below conventional thresholds and that alternative morphological indices, such as the inferior vena cava shape change index, may provide improved diagnostic performance compared with diameter alone [58].

In contrast, by directly assessing the hemodynamic consequences of elevated venous pressures at the organ level, VExUS improves the identification of clinically relevant congestion compared with isolated parameters. Comparative studies have shown that while simplified venous Doppler indices may offer higher sensitivity, integrated approaches such as VExUS provide greater specificity for predicting organ dysfunction and adverse outcomes [40].

Lung ultrasound (LUS) represents a complementary modality that specifically assesses pulmonary congestion [59]. The presence of B-lines reflects extravascular lung water and has been consistently associated with adverse outcomes in heart failure [60]. However, LUS primarily captures left-sided filling pressures and does not provide information on systemic venous congestion. Consequently, discordant patterns may occur, with patients exhibiting significant systemic congestion in the absence of pulmonary edema, or vice versa.

This distinction between pulmonary and systemic congestion is clinically relevant. Pulmonary congestion is predominantly driven by elevated left-sided pressures, whereas systemic congestion reflects right-sided hemodynamics and venous return impairment [61].

The dissociation between these compartments underscores the limitations of single-modality assessment and supports the need for integrated evaluation.

Recent studies have demonstrated that multimodal approaches combining different ultrasound techniques provide superior prognostic stratification compared with individual tools. In particular, the integration of VExUS with lung ultrasound significantly improves risk prediction, reflecting the multi-compartmental nature of congestion [30,62].

Furthermore, dynamic changes in pulmonary congestion, assessed by serial LUS, have been shown to carry important prognostic information, reinforcing the concept that congestion should be evaluated as a dynamic process rather than a static condition [25].

Biomarkers, including natriuretic peptides, remain essential for the diagnosis and risk stratification of heart failure. However, they primarily reflect myocardial wall stress and do not directly quantify venous congestion or its impact on organ function. Their levels may also be influenced by age, renal function, and comorbidities, limiting their specificity in the assessment of congestion [6].

Importantly, each modality captures a distinct compartment of congestion, and no single tool is sufficient to fully characterize its multi-organ nature. Collectively, these observations highlight a central limitation of traditional approaches: the risk of misclassification of congestion when relying on isolated parameters.

Patients may be categorized as decongested based on clinical examination or single-modality assessment while still harboring significant organ-level congestion. Conversely, abnormal findings in one compartment may not necessarily reflect clinically relevant congestion. VExUS represents a significant advancement by enabling the direct assessment of systemic venous congestion across multiple organ systems. However, its optimal use lies not in replacing existing tools, but in complementing them within a multimodal approach that integrates pulmonary, systemic, and hemodynamic information.

The physiological domains captured by different modalities used in congestion assessment are summarized in Table 3.

## 7. Clinical Applications in Heart Failure

The interpretation and clinical implications of VExUS should be considered within the context of the population being evaluated. Although the underlying physiological principle of venous pressure transmission is shared across different clinical settings, the prevalence, mechanisms, and prognostic significance of venous congestion may differ substantially among patients with acute heart failure, ambulatory heart failure, cardiorenal syndrome, dialysis-dependent populations, and heterogeneous critical care cohorts. Therefore, evidence derived from one setting should not be directly extrapolated to another without consideration of the specific clinical context.

### 7.1. Acute Heart Failure

In the setting of acute heart failure (AHF), early identification and quantification of congestion are essential for risk stratification and therapeutic decision-making [63,64]. Conventional assessment strategies often fail to detect subclinical or organ-level congestion, particularly in the early phases of hospitalization. In this context, VExUS and multimodal ultrasound approaches provide a more sensitive and physiologically grounded evaluation of systemic venous congestion.

Several prospective observational studies have demonstrated that higher degrees of venous congestion assessed by VExUS are associated with adverse clinical outcomes in acute heart failure. In a prospective study conducted in the emergency department setting, severe VExUS congestion was independently associated with increased risk of in-hospital mortality and early readmission [65].

Similarly, multimodal ultrasound studies integrating VExUS with pulmonary congestion assessment have consistently shown that patients with persistent venous congestion exhibit higher rates of rehospitalization and clinical deterioration [30].

In particular, severe congestion patterns have been linked to a markedly higher risk of in-hospital death and early readmission, outperforming traditional markers such as natriuretic peptides [65].

Similarly, VExUS grading has been shown to correlate with clinical severity and the need for more intensive decongestive therapy, reinforcing its role in early risk stratification [30]. Importantly, VExUS allows for the detection of congestion even in patients without overt clinical signs [33,37]. This is particularly relevant in the acute setting, where clinical examination may underestimate the true burden of congestion.

In intensive care unit settings, VExUS has also been applied to critically ill patients with hemodynamic instability. While its prognostic performance appears stronger in cardiac populations, studies suggest that dynamic changes in venous congestion during the early phase of hospitalization may provide clinically relevant information even in heterogeneous ICU cohorts [14]. This highlights the importance of serial assessment rather than reliance on single measurements [52]. However, it should be acknowledged that ICU populations differ substantially from patients hospitalized primarily for acute heart failure. In the ICU setting, organ dysfunction is often multifactorial, with sepsis, mechanical ventilation, vasopressor therapy, renal injury, and inflammatory or distributive mechanisms potentially coexisting with venous congestion. Consequently, both the prognostic performance and clinical interpretation of VExUS may not be directly extrapolated across these settings [65,66,67,68].

### 7.2. Cardiorenal Syndrome

The clinical relevance of venous congestion is particularly evident in the context of cardiorenal syndrome, where renal dysfunction is closely linked to elevated venous pressures [69]. Traditional interpretations have emphasized reduced cardiac output as the primary driver of renal impairment; however, accumulating evidence indicates that renal venous congestion plays a central role [70]. Importantly, the growing recognition of venous congestion should not be interpreted as excluding other pathophysiological pathways involved in cardiorenal syndrome. Contemporary models recognize cardiorenal dysfunction as the result of multiple interacting mechanisms, including reduced forward flow, neurohormonal activation, endothelial dysfunction, chronic kidney disease, elevated intra-abdominal pressure, and treatment-related factors. Venous congestion appears to be a particularly important and often underrecognized component of this complex pathophysiological network [71,72,73,74].

Ultrasound-based assessment of renal venous flow has provided key insights into this mechanism. Abnormal intrarenal Doppler patterns, reflecting impaired venous drainage, have been shown to correlate with the presence and severity of acute kidney injury, independently of cardiac output [20].

These findings support the concept that renal dysfunction in heart failure is largely driven by backward pressure transmission rather than forward flow limitation.

VExUS extends this concept by integrating renal venous assessment into a broader evaluation of systemic congestion. Studies in patients with cardiorenal syndrome have demonstrated that higher VExUS grades are associated with reduced diuretic efficiency and worsening renal function.

Conversely, improvement in venous congestion is associated with better renal outcomes, suggesting a direct pathophysiological link between congestion and kidney function [29].

The concept of congestive nephropathy emphasizes the importance of early detection. In many patients, renal dysfunction may represent a late manifestation of ongoing venous congestion [75]. Ultrasound-based markers, including intrarenal venous flow and VExUS grading, allow for the identification of early congestion-related alterations before changes in serum creatinine become apparent [32].

From a therapeutic perspective, this has important implications. Misinterpretation of renal dysfunction as a consequence of hypoperfusion may lead to inappropriate reduction in diuretic therapy, thereby perpetuating congestion [74]. In contrast, identifying venous congestion as the primary driver supports continued or intensified decongestive strategies, even in the presence of worsening renal function [33].

Nevertheless, caution is warranted when extrapolating findings from cardiorenal syndrome cohorts to the broader heart failure population, as the relative contribution of venous congestion to clinical outcomes may vary according to the severity of renal dysfunction, baseline kidney disease, and the presence of additional hemodynamic or non-hemodynamic determinants of renal injury [71,72].

### 7.3. Guiding Decongestive Therapy

One of the most promising applications of VExUS lies in its potential to guide decongestive therapy through dynamic assessment of congestion. Unlike static parameters, ultrasound-based evaluation allows for real-time monitoring of the response to treatment, enabling a more individualized and physiologically guided approach.

Several studies have demonstrated that changes in venous congestion over time are more informative than baseline measurements. In particular, reductions in VExUS grade during hospitalization have been associated with improved clinical outcomes, while persistent or worsening congestion is linked to higher risk of adverse events, supporting the use of serial VExUS assessment as a tool for monitoring treatment response [14,76].

The integration of VExUS into therapeutic decision-making may also improve the management of diuretic resistance. Venous congestion has been shown to impair renal sodium excretion and reduce the effectiveness of diuretic therapy, contributing to persistent volume overload [73]. By identifying patients with significant venous congestion, clinicians may optimize diuretic strategies or consider alternative approaches, such as ultrafiltration [77].

Furthermore, ultrasound-guided decongestion may help avoid both under- and over-treatment. In patients with persistent congestion, escalation of therapy may be warranted despite apparent clinical improvement. Conversely, normalization of venous flow patterns may indicate adequate decongestion, reducing the risk of excessive fluid removal and hemodynamic instability [78].

The role of VExUS in guiding fluid management extends beyond heart failure. Studies evaluating fluid challenges have shown that increases in venous congestion may occur without improvement in perfusion, highlighting the potential harm of indiscriminate fluid administration in patients with elevated venous pressures [79].

These findings reinforce the importance of assessing both congestion and perfusion when guiding therapy. The use of VExUS as a dynamic monitoring tool represents a shift toward a more precise and individualized approach to decongestive therapy, with the potential to improve both short- and long-term outcomes.

However, it should be emphasized that the current evidence supporting VExUS-guided therapeutic strategies remains largely observational. Although VExUS may facilitate the identification of residual venous congestion, monitor the response to decongestive therapies, and complement clinical decision-making, no randomized controlled trial has yet demonstrated that a VExUS-guided management strategy improves patient-centered outcomes compared with standard care. Therefore, VExUS should currently be regarded as an adjunctive tool for congestion assessment rather than a validated therapeutic target in itself [9,52,66].

### 7.4. Current Evidence and Clinical Implication

The growing interest in venous congestion and ultrasound-based assessment has led to an expanding body of literature evaluating the prognostic and clinical relevance of VExUS and related Doppler techniques. While the available evidence is predominantly observational, it provides consistent signals supporting the role of venous congestion as a key determinant of outcomes in heart failure [37,44].

Importantly, the available evidence is derived from clinically heterogeneous populations, including acute heart failure cohorts, ambulatory heart failure patients, cardiorenal syndrome populations, postoperative cardiac surgery cohorts, dialysis patients, and mixed ICU populations [37,47,72]. As a result, differences in study design, patient characteristics, and outcome definitions should be considered when interpreting the overall evidence base. The principal studies evaluating VExUS and related venous Doppler approaches across different clinical settings are summarized in Table 4.

Multiple cohort studies have demonstrated a strong association between venous congestion and adverse clinical outcomes. Across different clinical settings, higher VExUS grades have been consistently linked to increased mortality, rehospitalization, and organ dysfunction. In acute heart failure populations, severe congestion patterns are associated with markedly worse outcomes, even after adjustment for traditional risk factors [30,65].

Importantly, these associations extend beyond static measurements. Several studies have highlighted that dynamic changes in congestion carry greater prognostic significance than baseline values. Improvements in VExUS score during hospitalization have been associated with reduced mortality risk, whereas persistent or worsening congestion identifies patients at particularly high risk [14,76]. This finding has been reproduced across heterogeneous populations, including both cardiac and general ICU cohorts, suggesting that the trajectory of congestion is a key determinant of clinical evolution.

In addition to global VExUS grading, individual Doppler components also provide prognostic information. Abnormal portal vein pulsatility and intrarenal venous flow patterns have been associated with adverse outcomes, including in-hospital mortality and readmission [80].

However, studies comparing composite scores with individual parameters suggest that while single Doppler indices may be sensitive, integrated approaches such as VExUS improve specificity for clinically relevant congestion [81].

More recently, meta-analytic evidence has further supported the prognostic relevance of venous congestion assessment. A systematic review and meta-analysis evaluating VExUS and acute kidney injury reported that higher VExUS grades were associated with a significantly increased risk of AKI, with pooled effect estimates suggesting an approximately 2- to 3-fold higher risk of renal injury in patients with severe venous congestion [13]. However, substantial heterogeneity across studies was observed, reflecting differences in patient populations, ultrasound protocols, and clinical settings.

These findings reinforce the pathophysiological link between venous congestion and renal dysfunction observed in individual studies, but the strength of the association appears to vary across clinical contexts.

The predictive value of VExUS is more pronounced in populations with primary cardiac dysfunction, whereas its performance is less consistent in heterogeneous intensive care unit (ICU) cohorts, where multiple competing mechanisms contribute to organ injury. This variability underscores the importance of clinical context when interpreting congestion-related findings. The relationship between venous congestion and renal dysfunction is one of the most consistently reported findings in the literature. Both individual studies and meta-analytic data support a strong association between elevated venous pressures and the development of acute kidney injury. Intrarenal Doppler abnormalities and higher VExUS grades have been linked to increased incidence and severity of AKI, reinforcing the concept of congestion-driven renal impairment [13,20].

Importantly, this association appears to be largely independent of cardiac output, further supporting the role of backward failure as a key mechanism in cardiorenal syndrome.

Another important aspect of the evidence relates to the response to decongestive therapy. Studies evaluating serial ultrasound assessments have shown that reductions in venous congestion are associated with improved outcomes, whereas persistent congestion identifies patients at risk of treatment failure. In this regard, ultrasound-based monitoring may provide a more accurate reflection of therapeutic efficacy compared with conventional clinical or laboratory parameters.

The available evidence supports several consistent conclusions. First, venous congestion is a major determinant of clinical outcomes in heart failure, particularly in relation to renal dysfunction and rehospitalization.

Second, serial congestion assessment may provide clinically relevant information regarding treatment response and disease evolution.

Third, multimodal and integrative approaches outperform isolated parameters, reflecting the multi-organ nature of congestion.

However, important limitations must be acknowledged. The majority of studies are observational and relatively small, with significant heterogeneity in patient populations, ultrasound protocols, and outcome definitions. Randomized controlled trials evaluating VExUS-guided management strategies are currently lacking, limiting the ability to draw definitive conclusions regarding causality and therapeutic impact.

Despite these limitations, the consistency of findings across studies and clinical settings provides a strong rationale for incorporating venous congestion assessment into the clinical evaluation of HF. Nevertheless, the available evidence primarily supports the role of VExUS as a diagnostic, pathophysiological, and prognostic tool [37,47,52]. Whether systematic VExUS-guided management strategies translate into improved patient outcomes remains an important unanswered question that requires dedicated randomized clinical trials.

## 8. Limitation of VExUS

Despite the growing interest in VExUS and venous Doppler assessment, several methodological, technical, interpretative, and clinical limitations must be acknowledged before widespread implementation in routine practice.

First, VExUS is inherently operator-dependent. The acquisition and interpretation of Doppler waveforms (particularly at the level of the hepatic, portal, and intrarenal veins) require specific technical expertise and adequate acoustic windows. Variability in operator experience may affect both image quality and diagnostic accuracy, limiting reproducibility across different clinical settings [4,11]. This issue is particularly relevant for intrarenal Doppler assessment, which may be technically challenging and not feasible in all patients.

Closely related to this is the issue of interobserver variability. Although some components of ultrasound assessment, such as lung ultrasound, have demonstrated high reproducibility, Doppler-based venous assessment is more complex and subject to interpretation. Differences in waveform classification and grading may lead to inconsistencies in VExUS scoring, particularly in borderline cases.

Another key limitation is the lack of standardization. While the original VExUS grading system provides a structured approach, variations in acquisition techniques, definitions of abnormal patterns, and thresholds for severity have been reported across studies.

In addition, standardized training pathways, competency assessment, and reproducible acquisition protocols for multi-organ venous Doppler evaluation remain incompletely defined, potentially limiting broader implementation and inter-center reproducibility.

In some cases, simplified or modified versions of the score have been used, further contributing to heterogeneity and limiting comparability between studies [50]. Furthermore, VExUS does not directly measure venous pressure, but rather infers congestion from flow patterns, which may be influenced by multiple hemodynamic factors. This distinction is critical when interpreting its findings and integrating them into clinical decision-making.

From an evidence standpoint, the current literature is predominantly observational. Although consistent associations between venous congestion and adverse outcomes have been reported, observational studies cannot establish whether modifying management according to VExUS findings directly improves outcomes. At present, VExUS should therefore be regarded primarily as a tool for congestion assessment, physiological characterization, and risk stratification. The absence of randomized controlled trials evaluating VExUS-guided treatment strategies remains one of the most important evidence gaps in the field [1,37,82].

The applicability of VExUS outside specific clinical contexts also deserves consideration. While its performance appears robust in patients with heart failure and cardiorenal syndrome, results are less consistent in heterogeneous ICU populations, where multiple competing mechanisms contribute to organ dysfunction. In such settings, venous congestion may represent only one of several pathophysiological factors, reducing the predictive value of VExUS [14].

Several confounding clinical conditions may affect the interpretation of venous Doppler findings. Significant tricuspid regurgitation, pulmonary hypertension, and right ventricular dysfunction can alter venous flow patterns independently of congestion severity. Similarly, mechanical ventilation and changes in intrathoracic pressure may influence Doppler waveforms, potentially leading to misinterpretation [83].

An additional conceptual limitation relates to the interpretation of VExUS as a surrogate of volume status. Emerging data suggest that VExUS may reflect underlying cardiac dysfunction and venous pressure transmission rather than absolute intravascular volume. In particular, studies have shown that VExUS grading does not necessarily correlate with traditional markers of fluid overload, such as body weight, and may instead be more closely linked to ventricular function [50].

This distinction is important, as it challenges the assumption that congestion can be equated with volume excess.

Finally, the feasibility of comprehensive VExUS assessment in routine clinical practice may be limited by time constraints and resource availability. Full evaluation requires the acquisition of multiple Doppler signals, which may not be practical in all clinical settings, particularly in emergency or high-acuity environments [52].

This has led to interest in simplified approaches focusing on selected components, although the trade-off between feasibility and accuracy remains to be fully defined.

From a practical perspective, these limitations reinforce the need to interpret VExUS within a comprehensive multimodal assessment. VExUS findings should be integrated with echocardiographic evaluation of cardiac structure and function, lung ultrasound, natriuretic peptide levels, renal function, urine output, physical examination, and the overall response to therapy. Rather than serving as an isolated diagnostic tool, VExUS is most appropriately used as one component of a broader physiological assessment aimed at characterizing congestion and guiding clinical decision-making [44,47,52].

A careful understanding of its technical constraints, clinical context, and interpretative nuances is essential to avoid misapplication and to maximize its potential clinical utility.

## 9. Conclusions and Future Directions

The assessment of congestion in heart failure is undergoing a conceptual transformation. One of the central messages emerging from the available literature is that congestion should be interpreted not only according to fluid accumulation but also according to its impact on organ function. This organ-centered perspective provides a conceptual framework that may help integrate Doppler-based venous assessment, cardiorenal interactions, and multimodal ultrasound findings into a more clinically meaningful evaluation of congestion. Traditional approaches, largely centered on the estimation of intravascular volume, fail to capture the complexity of venous congestion and its impact on organ function. The emerging evidence reviewed in this manuscript supports a shift toward a physiology-based model in which congestion is defined by elevated venous pressures, impaired organ drainage, and their interaction with systemic perfusion. The emerging evidence reviewed in this manuscript supports a more integrative physiology-based model in which congestion results from the interaction between intravascular volume burden, venous pressure transmission, impaired organ drainage, neurohormonal activation, and systemic perfusion.

Within this evolving paradigm, ultrasound-based techniques and, in particular, the VExUS score offer a unique opportunity to directly visualize and quantify systemic venous congestion at the bedside. By integrating information from multiple venous territories, VExUS provides a more comprehensive and organ-relevant assessment compared with conventional tools. Importantly, dynamic changes in congestion, rather than static measurements, appear to be the most informative markers of clinical evolution and therapeutic response [14].

The clinical implications of this approach are substantial. Venous congestion has emerged as a key determinant of adverse outcomes, particularly in relation to renal dysfunction and heart failure rehospitalization. The recognition of congestion as a dynamic and multi-organ process opens the door to more precise and individualized management strategies, including the use of serial ultrasound assessment to guide decongestive therapy.

However, several challenges remain. The current evidence base is largely observational, and the lack of randomized trials limits the ability to establish causality and define standardized therapeutic algorithms. Technical complexity, operator dependency, and variability in acquisition and interpretation also represent important barriers to widespread implementation. Moreover, the role of VExUS must be considered within a broader clinical context, as its performance may vary across different patient populations and clinical scenarios.

Although the available data support the prognostic and physiological relevance of venous congestion assessment, current evidence is insufficient to recommend VExUS-guided treatment algorithms as standard care. At present, VExUS should be considered a complementary tool that may inform clinical decision-making when interpreted within a comprehensive multimodal assessment.

Future research should focus on several key areas. First, randomized controlled trials are needed to evaluate whether VExUS-guided management strategies translate into improved clinical outcomes. Second, efforts toward standardization of acquisition protocols and interpretation criteria are essential to enhance reproducibility and facilitate broader adoption. Third, the integration of VExUS with other modalities (such as lung ultrasound, biomarkers, and flow-based parameters) should be further explored to develop comprehensive, multimodal congestion assessment strategies.

Technological advances may also play a role in overcoming current limitations. The development of automated or artificial intelligence-assisted Doppler analysis has the potential to reduce operator dependency and improve consistency, making advanced ultrasound techniques more accessible in routine clinical practice [84,85].

In conclusion, VExUS should be viewed as a complementary tool that enhances, rather than replaces, existing methods of congestion assessment. Its greatest value lies in its ability to shift the clinical focus from fluid quantity to venous pressure and organ perfusion, enabling earlier detection of clinically relevant congestion, improved risk stratification and more targeted therapeutic interventions.

## Figures and Tables

**Table 1:** Traditional vs. Contemporary Conceptualization of Congestion in Heart Failure.

Domain	Traditional View	Contemporary (VExUS-Based) View
Definition of congestion	Fluid overload	Venous pressure–driven hemodynamic state
Pathophysiological driver	Intravascular volume excess	Elevated venous pressure and impaired organ drainage
Primary mechanism	Forward failure (low cardiac output)	Backward failure (venous congestion)
Organ involvement	Global/systemic	Organ-specific (renal, hepatic, splanchnic, pulmonary)
Key determinant of dysfunction	Reduced perfusion	Reduced perfusion gradient (arterial inflow − venous pressure)
Clinical assessment	Physical exam, weight, CVP	Multimodal ultrasound (VExUS, LUS, Doppler)
Nature of congestion	Static	Dynamic and evolving
Prognostic relevance	Based on symptoms/signs	Based on organ-level congestion and its trajectory
Therapeutic target	Volume removal	Reduction in venous pressure and restoration of organ perfusion

Abbreviations: CVP: central vein pressure; LUS: lung ultrasound; VExUS: venous excess ultrasound.

**Table 2:** VExUS Protocol: Technical Components and Doppler Interpretation. Abbreviations: IVC, inferior vena cava; RAP, right atrial pressure; VExUS, venous excess ultrasound; S, systolic; D, diastolic.

Component	Normal Pattern	Moderate Congestion	Severe Congestion	Physiological Meaning
IVC	≤2 cm, normal variation	>2 cm and inspiratory collapsibility >50%	Markedly dilated and inspiratory collapsibility < 50%	Elevated RAP
Hepatic veins	S > D	S < D	S wave reversal	Direct transmission of RAP
Portal vein	Continuous flow (<30% pulsatility)	Pulsatility 30–50%	Pulsatility > 50%	Loss of splanchnic compliance
Intrarenal veins	Continuous monophasic flow	Biphasic/discontinuous	Monophasic diastolic only	Renal venous congestion and impaired drainage
Integrated VExUS score	Normal IVC or normal Doppler	Mild Doppler abnormalities	Severe abnormalities in ≥1 territory	Organ-level congestion severity

**Table 3:** Clinical Interpretation Approach. Abbreviations: BNP: B-type Natriuretic Peptide; JVD: jugular vein distension; LUS: lung ultrasound; NT-proBNP: NT-proBNP **=** N-terminal pro–B-type Natriuretic Peptide;VExUS: venous excess ultrasound.

Modality	What It Assesses	Physiological Domain	Strengths	Limitations
**Clinical** **examination**	Peripheral edema, JVD, rales	Late systemic/pulmonary congestion	Widely available, rapid	Low sensitivity, poor reproducibility
**Biomarkers (BNP/NT-proBNP)**	Myocardial wall stress	Cardiac pressure/volume load	Diagnostic and prognostic value	Not specific for venous congestion
**IVC ultrasound**	Central venous pressure	Right atrial pressure	Simple, rapid	Does not reflect organ-level congestion
**Lung ultrasound (LUS)**	B-lines (extravascular lung water)	Pulmonary congestion	Sensitive, reproducible	Does not assess systemic congestion
**Intrarenal Doppler**	Venous flow patterns	Renal congestion	Early marker of organ dysfunction	Technically challenging
**Portal/hepatic Doppler**	Venous pulsatility	Splanchnic/hepatic congestion	Reflects systemic venous pressure transmission	Operator-dependent
**VExUS score**	Multi-organ venous Doppler integration	Systemic venous congestion	Integrates organ-level physiology, higher specificity	Complexity, lack of standardization
**Multimodal approach (VExUS + LUS ± flow)**	Combined congestion and perfusion	Global hemodynamic profile	Improved risk stratification	Requires expertise and integration

**Table 4:** Key clinical studies evaluating venous congestion and ultrasound-based congestion assessment across different clinical settings. Abbreviations: AKI: acute kidney injury; HF: heart failure; HfrEF: heart failure with reduced ejection fraction; ICU: intensive care unit; MAKE: major adverse kidney events; RAP: right atrial pressure; RVSI: renal venous stasis index; VExUS: venous excess ultrasound.

Study	Population (N)	Clinical Setting	Ultrasound Approach	Main Outcome(s)	Key Findings
Longino et al., 2024 [12]	81	Mixed cardiac population undergoing invasive hemodynamic assessment	VExUS	Right atrial pressure (RAP)	VExUS strongly correlated with RAP and demonstrated excellent discrimination for elevated RAP, supporting its physiological validity.
Landi et al., 2024 [65]	50	Acute HF (Emergency Department)	VExUS	In-hospital mortality, readmission	Severe VExUS congestion was independently associated with mortality and early readmission.
Anastasiou et al., 2024 [10]	175	Acute HF	VExUS	Congestion severity and outcomes	Multi-organ congestion assessed by VExUS was associated with adverse clinical outcomes and incremental prognostic information.
Campos-Sáenz de Santamaría et al., 2025 [44]	100	Hospitalized HF	VExUS + multimodal ultrasound	Clinical outcomes	Multimodal congestion assessment improved risk stratification and identified patients with persistent organ congestion despite apparent clinical stabilization.
Kobalava et al., 2024 [62]	207	Acute decompensated HF	VExUS + lung ultrasound	Death and rehospitalization	Combined venous and pulmonary congestion assessment provided superior prognostic stratification compared with individual modalities.
Lozano-Jiménez et al., 2026 [3]	249	Acute HF at discharge	Venous congestion ultrasound assessment	Rehospitalization and mortality	Residual subclinical congestion at discharge was associated with worse post-discharge outcomes despite clinical stability.
Hassan et al., 2025 [36]	109	Ambulatory HFrEF	VExUS	Death/HF hospitalization	Higher VExUS grades were associated with increased risk of worsening HF and short-term adverse outcomes.
Trpkov et al., 2021 [20]	21 ADHF/21 ACS controls	Cardiorenal syndrome	Intrarenal venous Doppler (RVSI)	AKI severity	Renal venous congestion strongly correlated with AKI severity and supported the concept of congestion-driven renal dysfunction.
Trigkidis et al., 2024 [14]	89	General ICU	Serial VExUS	MAKE30	Dynamic changes in VExUS (ΔVExUS), rather than single measurements, were independently associated with adverse renal outcomes.

## Data Availability

No new data were created.

## Abbreviations

The following abbreviations are used in this manuscript:

AHF	acute heart failure
AKI	acute kidney injury
CVP	central venous pressure
e-VExUS	extended venous excess ultrasound
HF	heart failure
ICU	intensive care unit
IVC	inferior vena cava
LUS	lung ultrasound
POCUS	point-of-care ultrasound
VExUS	venous excess ultrasound

## References

[1] McDonagh T.A., Metra M., Adamo M., Gardner R.S., Baumbach A., Böhm M., Burri H., Butler J., Čelutkienė J., Chioncel O. (2023). 2023 Focused Update of the 2021 ESC Guidelines for the diagnosis and treatment of acute and chronic heart failure. Eur. Heart J..

[2] Rubio-Gracia J., Demissei B.G., Ter Maaten J.M., Cleland J.G., O’Connor C.M., Metra M., Ponikowski P., Teerlink J.R., Cotter G., Davison B.A. (2018). Prevalence, predictors and clinical outcome of residual congestion in acute decompensated heart failure. Int. J. Cardiol..

[3] Lozano-Jiménez S., García Sebastian C., Vela Martín P., García Magallón B., Martín Centellas A., de Castro D., Mitroi C., del Prado Dìaz S., Hernàndez-Pèrez F., Jimènez-Blanco Bravo M. (2026). Prevalence and prognostic impact of subclinical venous congestion in patients hospitalized for acute heart failure. Eur. Heart J. Acute Cardiovasc. Care.

[4] Velasco Malagón S., Acosta-Gutiérrez E., Nuñez-Ramos J.A., Salinas S., Mora Pabón G. (2024). Subclinical Congestion Evaluated by Point of Care Ultrasound (POCUS) at Discharge Predicts Readmission in Patients with Acute Heart Failure: Prognostic Cohort Study. POCUS.

[5] Ganapathy A., Dungu J.N., Dimarco A.D., Madu A., Li B., Langley S., Watters P., Savage H.O. (2026). Advances in Congestion Assessment in Decompensated Heart Failure. Card. Fail. Rev..

[6] Di Fiore V., Del Punta L., De Biase N., Pellicori P., Gargani L., Dini F.L., Armenia S., Li Vigni M., Maremmani D., Masi S. (2025). Integrative assessment of congestion in heart failure using ultrasound imaging. Intern. Emerg. Med..

[7] Wainstein M.V., Machado G.P., Telo G.H., Silveira A.D.D., Nasi L.A., Araujo G.N.D. (2025). Point-of-care ultrasound in the evaluation of STEMI patients. Front. Cardiovasc. Med..

[8] Argaiz E.R., Rola P., Haycock K.H., Verbrugge F.H. (2022). Fluid management in acute kidney injury: From evaluating fluid responsiveness towards assessment of fluid tolerance. Eur. Heart J. Acute Cardiovasc. Care.

[9] Giangregorio F., Centenara E., Mazzocchi S., Gerra L., Tursi F., Imberti D., Aschieri D. (2025). Assessing Venous Congestion in Acute and Chronic Heart Failure: A Review of Splanchnic, Cardiac and Pulmonary Ultrasound: Part 1: Conventional B-Mode, Colordoppler, and Vexus Protocol. J. Clin. Med..

[10] Anastasiou V., Peteinidou E., Moysidis D.V., Daios S., Gogos C., Liatsos A.C., Didagelos M., Gossios T., Efthimiadis G.K., Karamitsos T. (2024). Multiorgan Congestion Assessment by Venous Excess Ultrasound Score in Acute Heart Failure. J. Am. Soc. Echocardiogr..

[11] Antit S., Romdhane S., Mtiri I., Zakhama L. (2025). The Role of Venous Excess Ultrasound Score in Optimizing Acute Heart Failure Diagnosis and Prognosis. J. Saudi Heart Assoc..

[12] Longino A., Martin K., Leyba K., Siegel G., Thai T.N., Riscinti M., Douglas I., Gill E., Burke J. (2024). Prospective Evaluation of Venous Excess Ultrasound for Estimation of Venous Congestion. Chest.

[13] Melo R.H., Gioli-Pereira L., Lourenço I.D., Da Hora Passos R., Bernardo A.T., Volpicelli G. (2025). Diagnostic accuracy of multi-organ point-of-care ultrasound for pulmonary embolism in critically ill patients: A systematic review and meta-analysis. Crit. Care.

[14] Trigkidis K.K., Siempos I.I., Kotanidou A., Zakynthinos S., Routsi C., Kokkoris S. (2024). Early Trajectory of Venous Excess Ultrasound Score Is Associated with Clinical Outcomes of General ICU Patients. Shock.

[15] Miller W.L. (2016). Fluid Volume Overload and Congestion in Heart Failure: Time to Reconsider Pathophysiology and How Volume Is Assessed. Circ. Heart Fail..

[16] Castro R., Kattan E., Retamal J., Hernández G., Pinsky M.R. (2025). Venous congestion from a vascular waterfall perspective: Reframing congestion as a dynamic Starling resistor phenomenon. ICMx.

[17] Gelman S., Warner D.S., Warner M.A. (2008). Venous Function and Central Venous Pressure: A Physiologic Story. Anesthesiology.

[18] Patsalis N., Kreutz J., Chatzis G., Syntila S., Choukeir M., Schieffer B., Markus B. (2024). Early risk predictors of acute kidney injury and short-term survival during Impella support in cardiogenic shock. Sci. Rep..

[19] Ehrman R.R., Sherwin R.L., Reynolds C.A., Korzeniewski S.J., Welch R.D., Kline J.A., Ying H., Levy P.D. (2025). Impact of Venous CONgestion on Organ Function and Outcomes in Sepsis (ICON-Sepsis): A prospective observational cohort study protocol. BMJ Open.

[20] Trpkov C., Grant A.D.M., Fine N.M. (2021). Intrarenal Doppler Ultrasound Renal Venous Stasis Index Correlates with Acute Cardiorenal Syndrome in Patients with Acute Decompensated Heart Failure. CJC Open.

[21] Fallick C., Sobotka P.A., Dunlap M.E. (2011). Sympathetically Mediated Changes in Capacitance: Redistribution of the Venous Reservoir as a Cause of Decompensation. Circ. Heart Fail..

[22] Balan C., Morosanu B., Fodoroiu A., Dobre V., Dumitrache A., Barbulescu R.T., Valeanu L., Robu C., Boros C., Nica A. (2025). Decoding portal vein pulsatility: Hemodynamic determinants in a post-hoc analysis of a prospective observational trial. Ann. Intensive Care.

[23] Andrei S., Nguyen M., Bouhemad B., Guinot P.G. (2025). High VExUS grades are linked to cardiac function in general intensive care unit patients. Eur. Heart J. Acute Cardiovasc. Care.

[24] Scagliola R., Bonino B., Viazzi F., Balbi M., Ameri P. (2021). Relationship between venous congestion and systemic hypoperfusion in cardiorenal syndrome. Eur. Heart J..

[25] Harrison N.E., Ehrman R., Collins S., Desai A.A., Duggan N.M., Ferre R., Gargani L., Goldsmith A., Kapur T., Lane K. (2024). The prognostic value of improving congestion on lung ultrasound during treatment for acute heart failure differs based on patient characteristics at admission. J. Cardiol..

[26] Shirakabe A., Matsushita M., Shibata Y., Shighihara S., Nishigoori S., Sawatani T., Kiuchi K., Asai K. (2023). Organ dysfunction, injury, and failure in cardiogenic shock. J. Intensive Care.

[27] Puzzovivo A., Monitillo F., Guida P., Leone M., Rizzo C., Grande D., Ciccone M., Iacoviello M. (2018). Renal Venous Pattern: A New Parameter for Predicting Prognosis in Heart Failure Outpatients. J. Cardiovasc. Dev. Dis..

[28] Husain-Syed F., Gröne H.J., Assmus B., Bauer P., Gall H., Seeger W., Ghofrani A., Ronco C., Birk H.W. (2021). Congestive nephropathy: A neglected entity? Proposal for diagnostic criteria and future perspectives. ESC Heart Fail..

[29] Abu-Naeima E., Fatthy M., Shalaby M.A.A.S., Ayeldeen G., Verbrugge F.H., Rola P., Beaubien-Souligny W., Fayed A. (2025). Venous Excess Doppler ultrasound assessment and loop diuretic efficiency in acute cardiorenal syndrome. BMC Nephrol..

[30] Campos Sáenz De Santamaría A., Alcaine Otín A., Crespo Aznarez S., Josa Laorden C., Esterellas Sánchez L., Sánchez Marteles M., Horna V.G., Fiestas Z.A., López I.G., Gracia J.R. (2025). Multimodal analysis of congestion and prognostic utility of the VExUS protocol in hospitalized heart failure patients at a tertiary care hospital. Rev. Clínica Española (Engl. Ed.).

[31] Samad M., Malempati S., Restini C.B.A. (2023). Natriuretic Peptides as Biomarkers: Narrative Review and Considerations in Cardiovascular and Respiratory Dysfunctions. Yale J. Biol. Med..

[32] Barone R., Di Terlizzi V., Goffredo G., Paparella D., Brunetti N.D., Iacoviello M. (2024). Renal Arterial and Venous Doppler in Cardiorenal Syndrome: Pathophysiological and Clinical Insights. Biomedicines.

[33] Mullens W., Abrahams Z., Francis G.S., Sokos G., Taylor D.O., Starling R.C., Young J.B., Tang W.W. (2009). Importance of Venous Congestion for Worsening of Renal Function in Advanced Decompensated Heart Failure. J. Am. Coll. Cardiol..

[34] Kuwahara N., Honjo T., Sone N., Imanishi J., Nakayama K., Kamemura K., Iwahashi M., Ohta S., Kaihotsu K. (2023). Clinical impact of portal vein pulsatility on the prognosis of hospitalized patients with acute heart failure. World J. Cardiol..

[35] Haddadin R., Aboujaoude C., Trad G. (2024). Congestive Hepatopathy: A Review of the Literature. Cureus.

[36] Hassan A., Khalil A.A., Mostafa A., Yehia H. (2025). Venous Excess Ultrasound (VExUS) score and short-term outcomes in ambulatory patients with heart failure with reduced ejection fraction: An exploratory study. Egypt. Heart J..

[37] Ponor C.G., Cepoi M.R., Spiridon M.R., Tudorancea I., Bobu A.M., Badescu M.C., Costache A.D., Cucută S., Costache-Enache I.I. (2026). Assessment of Congestion in Heart Failure Using VExUS: Current Evidence, Limitations and Clinical Perspectives. Life.

[38] Koratala A., Ibrahim M., Gudlawar S. (2024). VExUS to Guide Ultrafiltration in Hemodialysis: Exploring a Novel Dimension of Point of Care Ultrasound. POCUS.

[39] Mukherjee M., Rudski L.G., Addetia K., Afilalo J., D’Alto M., Freed B.H., Gargani L., Grapsa J., Hassoun P., Hua L. (2025). Guidelines for the Echocardiographic Assessment of the Right Heart in Adults and Special Considerations in Pulmonary Hypertension: Recommendations from the American Society of Echocardiography. J. Am. Soc. Echocardiogr..

[40] Bhardwaj V., Samprathi A., Saha K., Orozco N., Hegde P., Nizamudin M., Chacko J., Varma M., Denault A.G.V., Rola P. (2025). Dual doppler dynamics: Integrating femoral venous doppler and VExUS for predicting organ dysfunction in acute heart failure. J. Anesth. Analg. Crit. Care.

[41] Magnino C., Omedè P., Avenatti E., Presutti D., Iannaccone A., Chiarlo M., Moretti C., Gaita F., Veglio F., Milan A. (2017). Inaccuracy of Right Atrial Pressure Estimates Through Inferior Vena Cava Indices. Am. J. Cardiol..

[42] Guo X., Tang M., Jiang X., Lin J., Chen W., Song Y. (2026). Ultrasound of the inferior vena cava for fluid therapy decisions: Strengths, limitations, and an integrated approach. J. Int. Med. Res..

[43] Soliman-Aboumarie H., Denault A.Y. (2023). How to assess systemic venous congestion with point of care ultrasound. Eur. Heart J.-Cardiovasc. Imaging.

[44] Campos-Sáenz de Santamaría A., Albines Fiestas Z.S., Crespo-Aznarez S., Esterellas-Sánchez L.K., Sánchez-Marteles M., Garcés-Horna V., Josa-Laorden C., Alcaine-Otìn A., Gimenez-Lopez I., Rubio-Gracia J. (2025). VExUS Protocol Along Cardiorenal Syndrome: An Updated Review. J. Clin. Med..

[45] Piccione M.C., Colarusso L., Agricola E., Cameli M., De Luca A., Manganaro R., Barchitta A., D’Andrea A., Parato V., Trambaiolo P. (2025). How to Do Echo for Noninvasive Hemodynamic Evaluation of the Patient in the Intensive Care Unit: A Consensus Statement of the Italian Society of Echocardiography and Cardiovascular Imaging. J. Cardiovasc. Echogr..

[46] Nijst P., Martens P., Dupont M., Tang W.H.W., Mullens W. (2017). Intrarenal Flow Alterations During Transition from Euvolemia to Intravascular Volume Expansion in Heart Failure Patients. JACC Heart Fail..

[47] Rola P., Miralles-Aguiar F., Argaiz E., Beaubien-Souligny W., Haycock K., Karimov T., Dinh V.A., Spiegel R. (2021). Clinical applications of the venous excess ultrasound (VExUS) score: Conceptual review and case series. Ultrasound J..

[48] Guinot P.G., Longrois D., Andrei S., Nguyen M., Bouhemad B. (2024). Exploring congestion endotypes and their distinct clinical outcomes among ICU patients: A post-hoc analysis. Anaesth. Crit. Care Pain Med..

[49] Muñoz F., Born P., Bruna M., Ulloa R., González C., Philp V., Mondaca R., Blanco J., Valenzuela E., Retamal J. (2024). Coexistence of a fluid responsive state and venous congestion signals in critically ill patients: A multicenter observational proof-of-concept study. Crit. Care.

[50] Wong A., Olusanya O., Watchorn J., Bramham K., Hutchings S. (2024). Utility of the Venous Excess Ultrasound (VEXUS) score to track dynamic change in volume status in patients undergoing fluid removal during haemodialysis—The ACUVEX study. Ultrasound J..

[51] Koratala A., Reisinger N. (2022). Venous Excess Doppler Ultrasound for the Nephrologist: Pearls and Pitfalls. Kidney Med..

[52] Assavapokee T., Rola P., Assavapokee N., Koratala A. (2024). Decoding VExUS: A practical guide for excelling in point-of-care ultrasound assessment of venous congestion. Ultrasound J..

[53] Guinot P.G., Bahr P.A., Andrei S., Popescu B.A., Caruso V., Mertes P.M., Berthoud V., Nguyen M., Bouhemad B. (2022). Doppler study of portal vein and renal venous velocity predict the appropriate fluid response to diuretic in ICU: A prospective observational echocardiographic evaluation. Crit. Care.

[54] Melo R.H., Wong A., Koratala A., Kattan E., Da Hora Passos R. (2026). Femoral vein Doppler ultrasound for assessing venous congestion and right heart function: A scoping review. ICMx.

[55] Palazzuoli A., Ruocco G., Pellicori P., Gargani L., Coiro S., Lamiral Z., Ambrosio G., Rastogi T., Girerd N. (2024). Multi-modality assessment of congestion in acute heart failure: Associations with left ventricular ejection fraction and prognosis. Curr. Probl. Cardiol..

[56] Cox E.G.M., Koster G., Baron A., Kaufmann T., Eck R.J., Veenstra T.C., Hiemstra B., Wong A., Kwee T.C., Tulleken J.E. (2020). Should the ultrasound probe replace your stethoscope? A SICS-I sub-study comparing lung ultrasound and pulmonary auscultation in the critically ill. Crit. Care.

[57] Jobs A., Vonthein R., König I.R., Schäfer J., Nauck M., Haag S., Fichera C., Stiermaier T., Ledwoch J., Schneider A. (2020). Inferior vena cava ultrasound in acute decompensated heart failure: Design rationale of the CAVA-ADHF-DZHK10 trial. ESC Heart Fail..

[58] Li L., Xu Y., Chen X., Huang W. (2026). Assessing venous congestion in critical illness: Advantages of the inferior vena cava shape change index over diameter. Ann. Intensive Care.

[59] Soldati G., Demi M., Demi L. (2019). Ultrasound patterns of pulmonary edema. Ann. Transl. Med..

[60] Gargani L., Girerd N., Platz E., Pellicori P., Stankovic I., Palazzuoli A., Pivetta E., Miglioranza M., Soliman-Aboumarie H., Agricola E. (2023). Lung ultrasound in acute and chronic heart failure: A clinical consensus statement of the European Association of Cardiovascular Imaging (EACVI). Eur. Heart J. Cardiovasc. Imaging.

[61] Grigore M., Nicolae C., Grigore A.M., Balahura A.M., Păun N., Uscoiu G., Verde I., Ilieșiu A. (2025). Contemporary Perspectives on Congestion in Heart Failure: Bridging Classic Signs with Evolving Diagnostic and Therapeutic Strategies. Diagnostics.

[62] Kobalava Z.D., Vladimirovna T.V., Kanatbekovich S.B., Aslanova R.S., Alekseevich L.A., Sergeevich N.I., Pavlovich S., Vatsik-Gorodetskaya M., Tabatabaei G., Al-Zakwani I. (2024). Prognostic Role of Ultrasound Diagnostic Methods in Patients with Acute Decompensated Heart Failure. Oman Med. J..

[63] Rossini R., Valente S., Colivicchi F., Baldi C., Caldarola P., Chiappetta D., Cipriani M., Ferlini M., Gasparetto N., Gilardi R. (2021). ANMCO POSITION PAPER: Role of intra-aortic balloon pump in patients with acute advanced heart failure and cardiogenic shock. Eur. Heart J. Suppl..

[64] Gheorghiade M., Filippatos G., De Luca L., Burnett J. (2006). Congestion in Acute Heart Failure Syndromes: An Essential Target of Evaluation and Treatment. Am. J. Med..

[65] Landi I., Guerritore L., Iannaccone A., Ricotti A., Rola P., Garrone M. (2024). Assessment of venous congestion with venous excess ultrasound score in the prognosis of acute heart failure in the emergency department: A prospective study. Eur. Heart J. Open.

[66] Beaubien-Souligny W., Galarza L., Buchannan B., Lau V.I., Adhikari N.K.J., Deschamps J., Charbonney E., Denault A., Wald R. (2023). Prospective Study of Ultrasound Markers of Organ Congestion in Critically Ill Patients with Acute Kidney Injury. Kidney Int. Rep..

[67] Melo R.H., Gioli-Pereira L., Melo E., Rola P. (2025). Venous excess ultrasound score association with acute kidney injury in critically ill patients: A systematic review and meta-analysis of observational studies. Ultrasound J..

[68] Song J., Chen G., Lai D., Zhong L., Fan H., Hu W., Wang M., Hu C., Chen W., Ming Z. (2025). Association between the Venous Excess Ultrasound (VExUS) score and acute kidney injury in critically ill patients with sepsis: A multicenter prospective observational study. Ann. Intensive Care.

[69] Damman K., Van Deursen V.M., Navis G., Voors A.A., Van Veldhuisen D.J., Hillege H.L. (2009). Increased Central Venous Pressure Is Associated with Impaired Renal Function and Mortality in a Broad Spectrum of Patients with Cardiovascular Disease. J. Am. Coll. Cardiol..

[70] Tamayo-Gutierrez A., Ibrahim H.N. (2022). The Kidney in Heart Failure: The Role of Venous Congestion. Methodist DeBakey Cardiovasc. J..

[71] Kumar U., Wettersten N., Garimella P.S. (2019). Cardiorenal Syndrome. Cardiol. Clin..

[72] Rangaswami J., Bhalla V., Blair J.E.A., Chang T.I., Costa S., Lentine K.L., Lerma E.V., Mezue K., Molitch M., Mullens W. (2019). Cardiorenal Syndrome: Classification, Pathophysiology, Diagnosis, and Treatment Strategies: A Scientific Statement from the American Heart Association. Circulation.

[73] Lim S.Y., Kim S. (2021). Pathophysiology of Cardiorenal Syndrome and Use of Diuretics and Ultrafiltration as Volume Control. Korean Circ. J..

[74] Mullens W., Damman K., Testani J.M., Martens P., Mueller C., Lassus J., Tang W.W., Skouri H., Verbrugge F.H., Orso F. (2020). Evaluation of Kidney Function Throughout the Heart Failure Trajectory—A Position Statement from the Heart Failure Association of the European Society of Cardiology. Eur. J. Heart Fail..

[75] Cops J., Mullens W., Verbrugge F.H., Swennen Q., De Moor B., Reynders C., Penders J., Achten R., Driessen A., Dendooven A. (2018). Selective abdominal venous congestion induces adverse renal and hepatic morphological and functional alterations despite a preserved cardiac function. Sci. Rep..

[76] Saadi M., Silvano G.P., Telo G.H., Scolari F.L., Machado G.P., Almeida R.F., Biolo A., Silveira A. (2024). VExUS score improvement is associated with better outcomes in acute decompensated heart failure. Eur. Heart J..

[77] Sovetova S., Charaya K., Erdniev T., Shchekochikhin D., Bogdanova A., Panov S., Plaksina N., Mutalieva E., Ananicheva N., Fomin V. (2024). Venous Excess Ultrasound Score Is Associated with Worsening Renal Function and Reduced Natriuretic Response in Patients with Acute Heart Failure. J. Clin. Med..

[78] Di Maria A., Siligato R., Bondanelli M., Fabbian F. (2024). Venous Doppler flow patterns, venous congestion, heart disease and renal dysfunction: A complex liaison. World J. Cardiol..

[79] Ruste M., Reskot R., Schweizer R., Mayet V., Fellahi J.L., Jacquet-Lagrèze M. (2024). Changes in portal pulsatility index induced by a fluid challenge in patients with haemodynamic instability and systemic venous congestion: A prospective cohort study. Ann. Intensive Care.

[80] Beaubien-Souligny W., Benkreira A., Robillard P., Bouabdallaoui N., Chassé M., Desjardins G., Lamarche Y., White M., Bouchard J., Denault A. (2018). Alterations in Portal Vein Flow and Intrarenal Venous Flow Are Associated with Acute Kidney Injury After Cardiac Surgery: A Prospective Observational Cohort Study. J. Am. Heart Assoc..

[81] Torres-Arrese M., Mata-Martínez A., Luordo-Tedesco D., García-Casasola G., Alonso-González R., Montero-Hernández E., Cobo-Marcos M., Sanchez-Sauce B., Cuervas-Mons V., Tung-Chen Y. (2023). Usefulness of Systemic Venous Ultrasound Protocols in the Prognosis of Heart Failure Patients: Results from a Prospective Multicentric Study. J. Clin. Med..

[82] Heidenreich P.A., Bozkurt B., Aguilar D., Allen L.A., Byun J.J., Colvin M.M., Deswal A., Drazner M.H., Dunlay S.M., Evers L.R. (2022). 2022 AHA/ACC/HFSA Guideline for the Management of Heart Failure: Executive Summary: A Report of the American College of Cardiology/American Heart Association Joint Committee on Clinical Practice Guidelines. J. Am. Coll. Cardiol..

[83] Hovgaard H.L., Nielsen R.R., Laursen C.B., Frederiksen C.A., Juhl-Olsen P. (2018). When appearances deceive: Echocardiographic changes due to common chest pathology. Echocardiography.

[84] Kim J., Maranna S., Watson C., Parange N. (2025). A scoping review on the integration of artificial intelligence in point-of-care ultrasound: Current clinical applications. Am. J. Emerg. Med..

[85] Yan L., Li Q., Fu K., Zhou X., Zhang K. (2025). Progress in the Application of Artificial Intelligence in Ultrasound-Assisted Medical Diagnosis. Bioengineering.

